# Twelve-month functional and structural outcomes of *Candida* endogenous endophthalmitis in immunocompetent patients: a longitudinal cohort study of an outbreak

**DOI:** 10.3389/fcimb.2026.1901253

**Published:** 2026-07-17

**Authors:** Xiaohan Zhang, Jinfeng Zhang, Xuesong Lin

**Affiliations:** 1 Ningde Clinical Medical College of Fujian Medical University, Department of Ophthalmology, Ningde Municipal Hospital, Ningde, China; 2 Shengli Clinical Medical College of Fujian Medical University, Department of Ophthalmology, Fuzhou University Affiliated Provincial Hospital, Fuzhou, China

**Keywords:** *Candida albicans*, endogenous endophthalmitis, iatrogenic outbreak, macular neovascularization, optical coherence tomography, retinal pigment epithelium

## Abstract

**Background:**

To characterize twelve-month visual outcomes, structural complications, and retinal pigment epithelium (RPE) dynamics stratified by initial lesion morphology in an iatrogenic, single-center outbreak of *Candida* endogenous endophthalmitis (EFE) among immunocompetent patients.

**Methods:**

This retrospective longitudinal cohort study included patients from a single-source outbreak. Of 26 eyes in the original cohort, 18 were classified with chorioretinal involvement and 8 with intraretinal or preretinal involvement. After excluding eyes with severe baseline destruction or loss to follow-up, 13 chorioretinal eyes and 8 intraretinal or preretinal eyes completed the twelve-month follow-up. Main outcome measures included best-corrected visual acuity (BCVA), complication rates, the incidence of secondary macular neovascularization (MNV) using optical coherence tomography angiography (OCTA), and longitudinal RPE lesion area changes quantified via optical coherence tomography (OCT).

**Results:**

Although both groups experienced functional improvement after treatment, the chorioretinal group had significantly worse final BCVA at twelve months (median 0.80 versus 0.20 LogMAR; *P* = 0.005) and a higher overall complication burden (92.3% versus 25.0%; *P* < 0.001) than the intraretinal or preretinal group. Secondary MNV developed in 100.0% of evaluable eyes in the chorioretinal group (10/10; 95% CI, 69.2%–100.0%), whereas no MNV occurred in the intraretinal/preretinal group (0/8; 95% CI, 0.0%–36.9%; *P* < 0.001). In the chorioretinal group, the RPE lesion area increased during the first three months and subsequently decreased by month twelve, occurring concurrently with sustained BCVA improvement.

**Conclusions:**

Initial chorioretinal involvement was associated with poorer visual recovery and a markedly higher incidence of secondary MNV. The early expansion of RPE lesions alongside visual improvement likely reflects tissue remodeling and may be less suggestive of ongoing infection. These findings support routine OCT and OCTA surveillance and timely anti-vascular endothelial growth factor therapy for eyes with chorioretinal involvement.

## Introduction

Endogenous fungal endophthalmitis (EFE) is a rare but devastating intraocular infection. Traditionally, EFE predominantly affects immunocompromised individuals or those with severe systemic comorbidities (e.g., critical illness, intravenous drug abuse, or immunosuppressive therapy) (Danielescu et al., 2020; Chen et al., 2022; Das et al., 2022; Burton et al., 2024). *Candida albicans* is the primary causative pathogen, often inducing severe chorioretinitis and vitreitis (Lingappan et al., 2012; Tanaka et al., 2016). Despite timely diagnosis and aggressive interventions, including pars plana vitrectomy (PPV) and antifungal therapy, the overall visual prognosis frequently remains guarded (Schiedler et al., 2004; Zhang and Wang, 2005; Shen and Xu, 2009).

However, the clinical spectrum and long-term sequelae of EFE in immunocompetent individuals remain largely understudied, as such cases typically only arise from specific iatrogenic contamination (Gupta et al., 2000; Dogra et al., 2018; Karkhur et al., 2020). We recently reported an outbreak of infusion-related *Candida albicans* EFE among otherwise healthy patients exposed to contaminated intravenous fluids (Zhang et al., 2026). This single-source outbreak involving clonally related *Candida* isolates provided an opportunity to evaluate the natural history and ocular complications of *Candida* EFE with fewer confounding systemic comorbidities.

The depth of retinal and choroidal involvement may influence prognosis in various chorioretinal diseases (Stephens et al., 2017; Zhuang et al., 2020). However, its precise impact on long-term clinical trajectories in *Candida* EFE has not been well characterized. Specifically, the development of secondary MNV—a major cause of irreversible vision loss in inflammatory disorders (Bihaniya et al., 2024; Wang et al., 2025)—has not been thoroughly investigated regarding its incidence and risk factors within a strictly immunocompetent cohort.

While our previous report established the epidemiological source, clonal lineage, and short-term surgical outcomes of this rare outbreak (Zhang et al., 2026), several clinical and structural questions require further investigation. Specifically, the twelve-month natural history and functional prognosis of healthy individuals recovering from severe, culture-proven *Candida* EFE have not been fully characterized. In addition, the relationship between the initial anatomical depth of tissue involvement (chorioretinal versus intraretinal or preretinal) and the development of secondary MNV remains to be determined in a cohort of patients without systemic comorbidities.

To address these questions, we conducted a longitudinal cohort study representing a twelve-month follow-up of this outbreak. Using serial optical coherence tomography (OCT) and optical coherence tomography angiography (OCTA), we aimed to characterize the twelve-month visual trajectories, structural complications, and RPE dynamics according to the initial lesion morphology. These findings may provide a practical anatomical framework to guide risk stratification, long-term surveillance, and therapeutic intervention for EFE.

## Methods

### Study design and participants

This retrospective, single-center, longitudinal cohort study was approved by the institutional ethics committee of Ningde Municipal Hospital (Approval No. NSYKYLL-2025-36; written informed consent waived) and adhered to the Declaration of Helsinki. The study was conducted entirely at Ningde Municipal Hospital, a tertiary referral center where all affected patients from this specific outbreak were admitted, treated, and followed. The cohort comprised 26 eyes from a distinct outbreak of infusion-related *Candida albicans* EFE occurring between May and November 2024. The epidemiological investigation, microbiological confirmation of clonal isolates, and criteria used to confirm immunocompetent status (strict absence of systemic immunosuppression, HIV, or debilitating diseases) have been detailed previously (Zhang et al., 2026). Eyes were followed for up to 12 months to evaluate lesion-pattern-based visual outcomes, RPE lesion-area changes, and complications.

### Eye selection and initial lesion-pattern classification

Initial lesion patterns were independently graded by two retina specialists (S.C., Y.W.; Cohen’s kappa = 0.92) using pre-defined objective criteria. In this study, “initial lesion morphology” refers to the earliest assessable lesion pattern determined from perioperative findings, including intraoperative observations and postoperative day-1 OCT, rather than from preoperative OCT alone. This approach was used because severe media opacity frequently precluded reliable preoperative OCT assessment, and intraretinal/preretinal lesions, such as fungal balls, were often removed or substantially reduced during PPV. Therefore, final classification was based on intraoperative observations together with postoperative day-1 OCT findings. In cases where a discrepancy arose between intraoperative vitrectomy findings and postoperative day-1 OCT observations regarding lesion depth, a final consensus classification was adjudicated by a senior vitreoretinal specialist (X.L.) through a joint review of the intraoperative surgical video and all sequential multimodal imaging.

Eyes were categorized into two groups:

1. Chorioretinal involvement: OCT evidence of outer retinal, RPE, or choroidal disruption, subretinal extension, and/or chorioretinal scarring.
2. Intraretinal/preretinal involvement: lesions confined to the inner retina or vitreoretinal interface, with postoperative day-1 OCT showing preserved outer retinal and RPE structures after surgical removal of vitreoretinal fungal material.

Macular involvement was further subclassified as topographic or structural. Topographic macular involvement was defined as infectious opacities suspended anterior to or overlying the macula without OCT-confirmed outer retinal/RPE/choroidal disruption. Structural macular involvement was defined as OCT-confirmed outer retinal, RPE, or choroidal disruption involving the fovea or parafovea. Eyes with severe pre-treatment intraocular destruction or loss to follow-up before 12 months were excluded from visual and complication analyses.

### Treatment and follow-up

All eyes underwent 25-gauge PPV, vitreous sampling, and intravitreal voriconazole injection. Importantly, none of the eyes received silicone oil tamponade during the primary PPV; all procedures were completed using air or balanced salt solution infusion. Furthermore, no patient underwent concurrent lensectomy or cataract surgery during the primary procedure, with the exception of one eye that required combined phacoemulsification due to a dense cataract that severely obscured the intraoperative view. Subsequently, patients received a standardized systemic voriconazole regimen (intravenous loading and maintenance, transitioning to oral therapy for four weeks). Additional intravitreal antifungal injections were administered based on clinical response. Follow-up visits occurred at baseline and months 3, 6, 9, and 12.

For the management of secondary complications, active secondary MNV was treated with intravitreal conbercept (0.5 mg/0.05 mL). Secondary MNV was diagnosed via OCTA, and treatment was initiated only in the presence of active exudative features (such as intraretinal and/or subretinal fluid) on structural B-scan OCT. Inactive MNV, characterized by a neovascular network on OCTA but lacking fluid or exudation, was monitored closely without intervention. The anti-VEGF treatment protocol consisted of a loading regimen of three consecutive monthly injections, followed by a pro re nata (PRN) retreatment scheme. Retreatment during the PRN phase was indicated if recurrent or persistent active exudation (intraretinal/subretinal fluid) was observed on serial structural OCT scans.

### Imaging assessment and outcome definitions

OCT and OCT angiography (OCTA) scans were acquired using the CIRRUS HD-OCT 5000 system (Carl Zeiss Meditec) equipped with FastTrac retinal tracking technology to minimize motion artifacts. Longitudinal RPE lesion area was primarily quantified using the built-in Advanced RPE Analysis software. To resolve automated segmentation errors caused by dense inflammatory debris, subretinal fluid, or tissue necrosis in the acute phase, all automated retinal layer segmentation lines on B-scans were systematically inspected by two independent masked retinal specialists. In cases of clear segmentation misalignment or automated identification failure, manual corrections were performed using the software’s built-in editing tools. For lesions where automated boundary detection was completely obscured, the spatial boundaries of RPE/Bruch’s membrane disruption were delineated manually using the built-in caliper and measurement tools within the Carl Zeiss proprietary analysis software (cross-referenced with corresponding en face OCTA and fundus images) to ensure consistency and comparability across visits. Secondary MNV was diagnosed via OCTA; active MNV required concurrent exudative features (intraretinal/subretinal fluid) on structural OCT, whereas inactive MNV lacked exudation.

The primary outcome was best-corrected visual acuity (BCVA, converted to LogMAR) at 12 months. Secondary outcomes included longitudinal BCVA changes, RPE lesion-area changes, and complication rates.

### Statistical analysis

Between-group baseline comparisons utilized the Mann–Whitney U test, Fisher’s exact test, or chi-square test. Longitudinal BCVA comparisons were performed using generalized estimating equation (GEE) models. Although the sample size was relatively small, GEE was employed as a necessary statistical adjustment to account for inter-eye correlation among patients with bilateral involvement, thereby minimizing type I error. Additionally, to visualize specific time-point differences, cross-sectional comparisons of BCVA between groups at each individual follow-up visit were conducted using the Mann-Whitney U test. For the chorioretinal group, temporal changes in RPE lesion area and BCVA were assessed using the Friedman test, followed by Wilcoxon signed-rank tests for planned *post hoc* paired comparisons (baseline vs. month 3; month 3 vs. month 12). *P* < 0.05 was considered statistically significant. In addition, within the chorioretinal group with evaluable longitudinal RPE measurements (n = 10), Spearman rank correlation was used to explore the relationship between RPE lesion area (baseline and month 3) and the final 12-month LogMAR BCVA.

## Results

### Study cohort and eye selection

**Figure 1:**
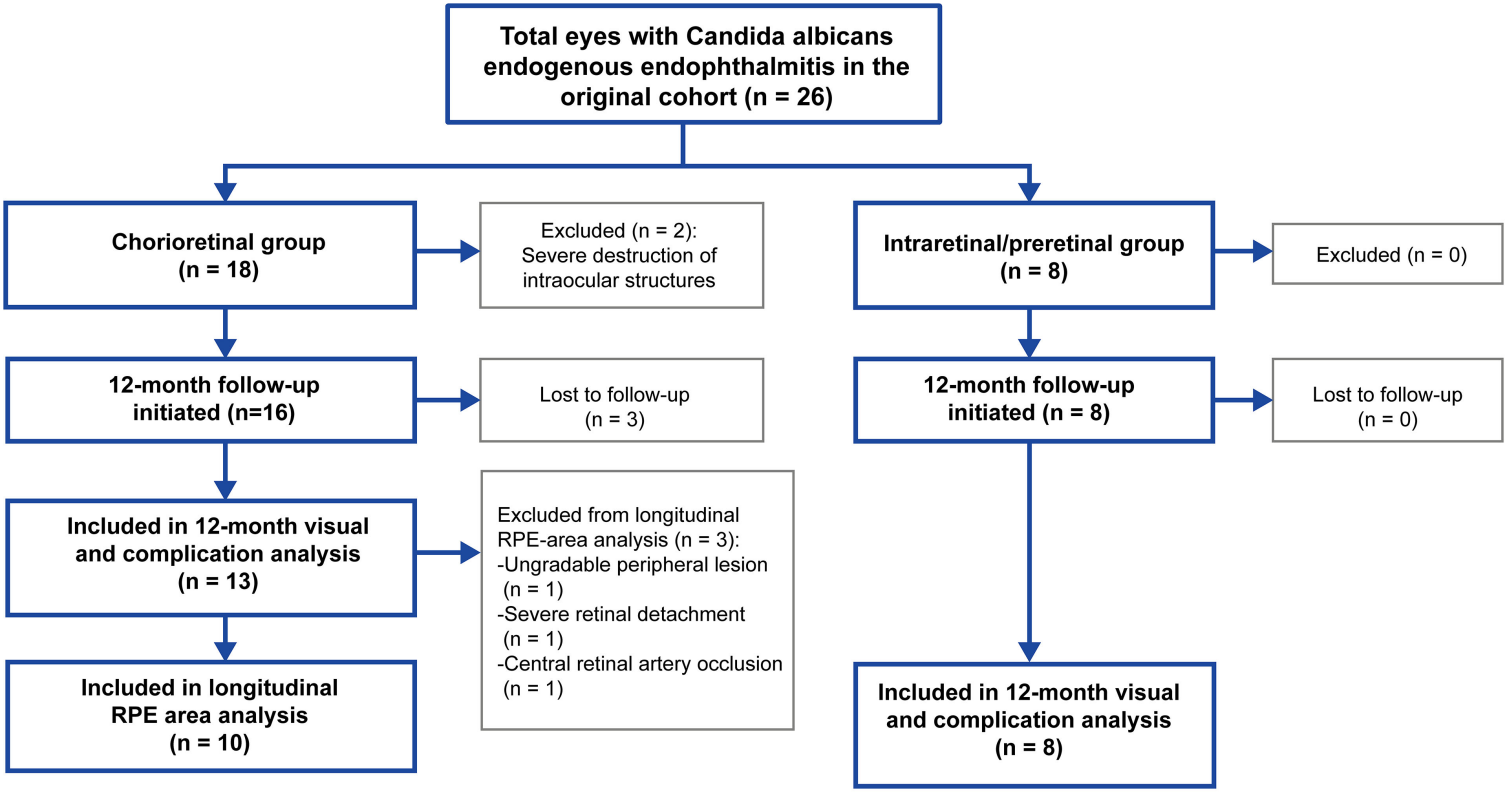
Flowchart of eye selection, grouping, and analysis. Of the 26 eyes in the original outbreak cohort, 18 were initially classified as chorioretinal involvement and 8 as intraretinal/preretinal involvement. After exclusion of eyes with severe baseline destruction and loss to follow-up, 13 and 8 eyes, respectively, were included in the 12-month visual and complication analyses. Ten chorioretinal eyes were evaluable for longitudinal RPE lesion-area analysis.

A total of 26 eyes with infusion-related *Candida albicans* EFE in immunocompetent patients were identified in the original cohort and categorized according to lesion pattern into the chorioretinal group (n = 18 eyes) and the intraretinal/preretinal group (n = 8 eyes). In the chorioretinal group, 2 eyes were excluded at baseline because of severe destruction of intraocular structures, and 3 eyes were lost to follow-up, leaving 13 eyes for the 12-month visual outcome and complication analyses. For the longitudinal RPE lesion-area analysis in this group, 3 additional eyes were excluded because of ungradable peripheral lesions (n = 1), severe retinal detachment (n = 1), or central retinal artery occlusion (n = 1), resulting in 10 evaluable eyes. In the intraretinal/preretinal group, all 8 eyes completed the 12-month follow-up and were included in the final visual outcome and complication analyses (**Figure 1**).

### Baseline characteristics

Baseline characteristics of eyes included in the 12-month outcome analysis are summarized in **Table 1**. The chorioretinal group comprised 13 eyes, and the intraretinal/preretinal group comprised 8 eyes. Age and sex distribution were comparable between the two groups. The median age was 50.0 years (interquartile range [IQR], 47.0–53.0 years) in the chorioretinal group and 52.0 years (IQR, 49.8–59.5 years) in the intraretinal/preretinal group (*P* = 0.191). Male sex accounted for 76.9% and 50.0% of eyes in the two groups, respectively (*P* = 0.346).

**Table 1:** Eye-level baseline characteristics by initial lesion pattern in immunocompetent patients with infusion-related *Candida albicans* endogenous endophthalmitis.

Variable	Chorioretinal group (n = 13 eyes)	Intraretinal/ Preretinal group (n = 8 eyes)	*P* value
Demographics			
Age, years	50.0 (47.0–53.0)	52.0 (49.8–59.5)	0.191
Male sex, n (%)	10 (76.9)	4 (50.0)	0.346
Baseline ocular status			
Baseline BCVA, LogMAR	2.0 (1.4–2.0)	0.4 (0.1–1.0)	0.057
IOP, mmHg	14.0 (11.0–15.0)	14.5 (13.8–15.2)	0.510
Topographic macular involvement, n (%)	4 (30.7)	3 (37.5)	1.000
Structural macular involvement, n (%)	12 (92.3)	0 (0.0)	<0.001
Hypopyon, n (%)	2 (15.4)	0 (0.0)	0.505
Vitreous haze ≥3+, n (%)	6 (46.2)	2 (25.0)	0.400
Management, microbiology, and timing			
Symptom onset to PPV, days	6.0 (5.0–8.0)	6.5 (5.8–19.2)	0.444
Presentation to PPV, days	0.0 (0.0–1.0)	0.0 (0.0–1.0)	1.000
Vitreous culture positive, n (%)	8 (61.5)	6 (75.0)	0.656
Prior oral corticosteroid exposure, n (%)	2 (15.4)	0 (0.0)	0.505
Intravitreal corticosteroid injection, n (%)	0 (0.0)	0 (0.0)	1.000

Continuous variables are presented as median (interquartile range), and categorical variables are presented as n (%). Between-group comparisons were performed using the Mann–Whitney U test for continuous variables and Fisher’s exact test for categorical variables. Because some patients contributed both eyes, eye-level baseline comparisons are exploratory; longitudinal outcomes were analyzed using generalized estimating equation models to account for inter-eye correlation. Vitreous haze was dichotomized as <3+ versus ≥3+. Topographic macular involvement indicates any lesion overlying the macula, whereas structural macular involvement denotes OCT-confirmed disruption of the macular outer retina, RPE, or choroid.

BCVA, best-corrected visual acuity; IOP, intraocular pressure; LogMAR, logarithm of the minimum angle of resolution; OCT, optical coherence tomography; PPV, pars plana vitrectomy; RPE, retinal pigment epithelium.

At baseline, eyes with chorioretinal involvement tended to have worse BCVA than eyes with intraretinal/preretinal involvement, although the difference did not reach statistical significance. The median baseline BCVA was 2.0 LogMAR (IQR, 1.4–2.0) in the chorioretinal group and 0.4 LogMAR (IQR, 0.1–1.0) in the intraretinal/preretinal group (*P* = 0.057). Baseline intraocular pressure was similar between groups, with median values of 14.0 mmHg (IQR, 11.0–15.0 mmHg) and 14.5 mmHg (IQR, 13.8–15.2 mmHg), respectively (*P* = 0.510).

Topographic macular involvement was present in 4 eyes (30.7%) in the chorioretinal group and 3 eyes (37.5%) in the intraretinal/preretinal group (*P* = 1.000). In contrast, structural macular involvement (affecting the outer retina/RPE/choroid) was present in 92.3% (12/13) of eyes in the chorioretinal group, whereas it was completely absent in the intraretinal/preretinal group (0/8 eyes, 0.0%; *P* < 0.001). Other baseline inflammatory findings, including hypopyon and vitreous haze ≥3+, did not differ significantly between groups. Hypopyon was observed in 2 eyes (15.4%) in the chorioretinal group and in none of the intraretinal/preretinal eyes (*P* = 0.505). Vitreous haze ≥3+ was present in 6 eyes (46.2%) and 2 eyes (25.0%), respectively (*P* = 0.400).

The interval from symptom onset to PPV was also comparable between groups, with a median of 6.0 days (IQR, 5.0–8.0 days) in the chorioretinal group and 6.5 days (IQR, 5.8–19.2 days) in the intraretinal/preretinal group (*P* = 0.444). The interval from presentation to PPV was 0.0 days (IQR, 0.0–1.0 days) in both groups (*P* = 1.000). Vitreous culture positivity rates were 61.5% and 75.0%, respectively (*P* = 0.656). Prior oral corticosteroid exposure was documented in 2 eyes (15.4%) in the chorioretinal group and in no eyes in the intraretinal/preretinal group (*P* = 0.505). No eye in either group had received intravitreal corticosteroid injection before treatment.

### Longitudinal visual outcomes

**Figure 2:**
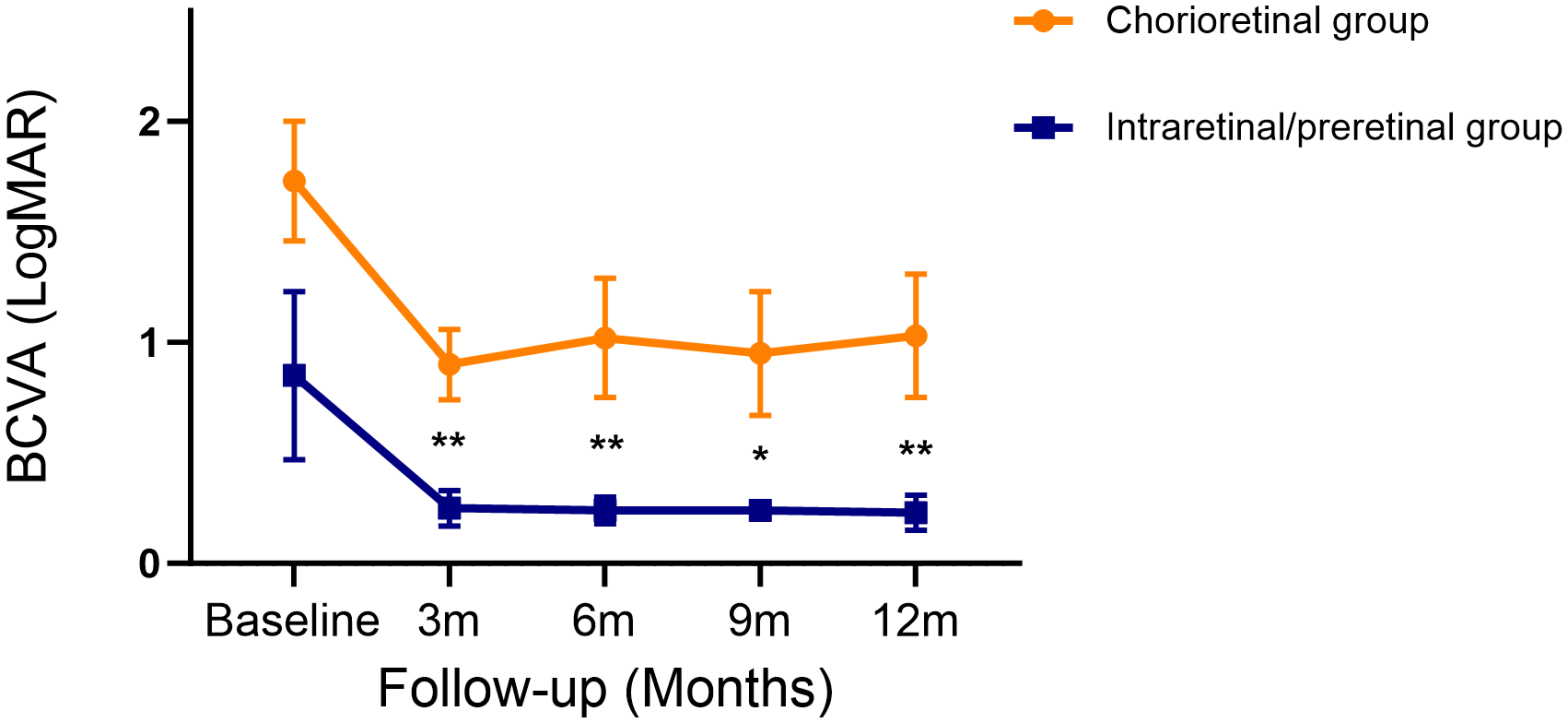
Longitudinal changes in best-corrected visual acuity over 12 months by lesion-pattern group. Mean best-corrected visual acuity (BCVA; LogMAR) is shown from baseline to month 12 for the chorioretinal group (n = 13 eyes) and the intraretinal/preretinal group (n = 8 eyes). Error bars indicate the standard error of the mean (SEM). Overall longitudinal between-group differences were assessed using generalized estimating equation (GEE) models to account for inter-eye correlation (overall *P* = 0.002). Asterisks indicate significant cross-sectional between-group differences at individual follow-up visits evaluated using the Mann-Whitney U test. The intraretinal/preretinal group showed lower LogMAR BCVA values, indicating better visual acuity, than the chorioretinal group at months 3, 6, 9, and 12. *P < 0.05; **P < 0.01.

Visual outcomes over 12 months are presented in **Table 2**, **Figure 2**. At 12 months after PPV, both groups showed improvement in median BCVA compared with baseline. In the chorioretinal group, median BCVA improved from 2.00 LogMAR (IQR, 1.40–2.00) at baseline to 0.80 LogMAR (IQR, 0.40–1.30) at 12 months. In the intraretinal/preretinal group, median BCVA improved from 0.40 LogMAR (IQR, 0.10–1.03) at baseline to 0.20 LogMAR (IQR, 0.10–0.23) at 12 months.

**Table 2:** Eye-level 12-month clinical outcomes and postoperative complications by initial lesion pattern.

Variables	Chorioretinal group (n = 13 eyes)	Intraretinal/ preretinal group (n = 8 eyes)	*P* value
Visual outcomes (LogMAR)			
Baseline BCVA	2.00 (1.40, 2.00)	0.40 (0.10, 1.03)	0.057
Final BCVA at 12 months	0.80 (0.40, 1.30)	0.20 (0.10, 0.23)	0.005
BCVA change	-0.70 (-1.50, -0.10)	-0.15 (-0.98, 0.03)	0.445
Postoperative complications			
Eyes with any postoperative complication**, n**/N **(%)** [95% CI]	12/13 (92.3) [66.7–98.6]	2/8 (25.0) [7.1–59.1]	<0.001
Vision-threatening complications			
Secondary MNV among evaluable eyes, n/N (%) [95% CI]	10/10 (100.0) [69.2–100.0]	0/8 (0.0) [0.0–36.9]	<0.001
Retinal detachment	1 (7.7)	0 (0.0)	1.000
Central retinal artery occlusion	1 (7.7)	0 (0.0)	1.000
Relapse of infection	0 (0.0)	1 (12.5)	0.381
Other ocular complications			
Anterior uveitis	5 (38.5)	1 (12.5)	0.354
Complicated cataract	1 (7.7)	1 (12.5)	1.000
PAS/secondary glaucoma	1 (7.7)	0 (0.0)	1.000

Data for visual outcomes are presented as median (interquartile range), and complications are presented as n (%) unless otherwise specified. BCVA change was calculated as final BCVA minus baseline BCVA; negative values indicate visual improvement. P values were calculated using the Mann–Whitney U test for continuous variables and Fisher’s exact test for categorical variables. For key proportions, 95% confidence intervals (95% CI) were calculated using the Wilson score method. Individual eyes may have experienced multiple overlapping or sequential complications during the 12-month follow-up period; therefore, the cumulative number of specific complication events may exceed the number of affected eyes. Secondary MNV was assessed among eyes with evaluable longitudinal retinal imaging; in the chorioretinal-involvement group, three eyes were excluded from MNV assessment because of severe confounding complications, including retinal detachment and central retinal artery occlusion, or ungradable peripheral lesions.

BCVA, best-corrected visual acuity; CI, confidence interval; MNV, macular neovascularization; PAS, peripheral anterior synechiae.

Despite visual improvement in both groups, final visual acuity remained significantly worse in the chorioretinal group than in the intraretinal/preretinal group at 12 months (median final BCVA, 0.80 vs. 0.20 LogMAR; *P* = 0.005). The median BCVA change, calculated as final BCVA minus baseline BCVA, was −0.70 LogMAR (IQR, −1.50 to −0.10) in the chorioretinal group and −0.15 LogMAR (IQR, −0.98 to 0.03) in the intraretinal/preretinal group, with no statistically significant between-group difference in magnitude of visual improvement (*P* = 0.445).

Overall longitudinal analysis using GEE models, accounting for inter-eye correlation, confirmed that the intraretinal/preretinal group maintained better visual acuity (lower LogMAR BCVA) than the chorioretinal group throughout the follow-up period (β = −0.880, 95% CI: −1.437 to −0.323, *P* = 0.002). Furthermore, point-by-point cross-sectional comparisons revealed that these between-group differences were statistically significant at individual visits at months 3, 6, 9, and 12 (**Figure 2**).

### Longitudinal changes in RPE lesion area and corresponding visual function

**Figure 3:**
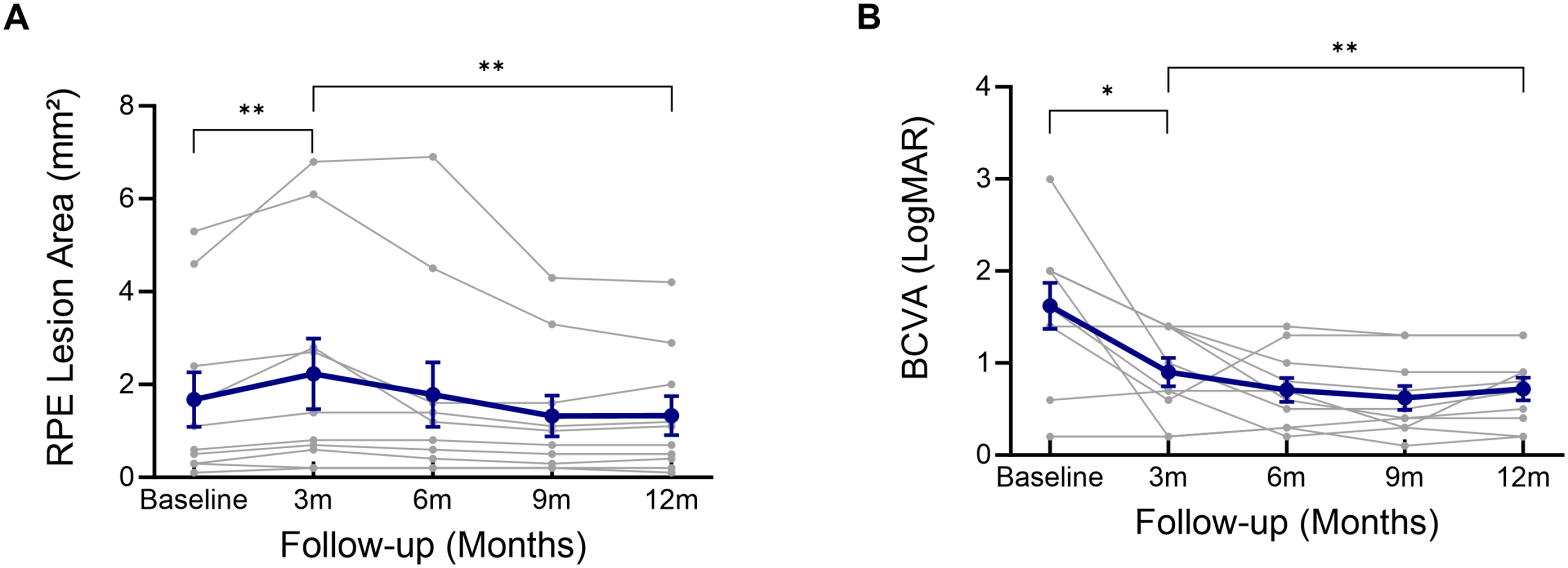
Longitudinal changes in retinal pigment epithelium lesion area and best-corrected visual acuity in the chorioretinal group. **(A)** Retinal pigment epithelium (RPE) lesion area and **(B)** best-corrected visual acuity (BCVA; LogMAR) were assessed over 12 months in eyes included in the longitudinal RPE-area analysis (n = 10 eyes). Bold blue lines represent mean values, and error bars indicate the standard error of the mean (SEM). Light gray lines with smaller dots represent individual eye-level trajectories. Overall changes across time were assessed using the Friedman test, followed by Wilcoxon signed-rank tests for paired comparisons. Reported pairwise *P* values were for baseline versus month 3 and month 3 versus month 12 (for RPE lesion area), and baseline versus month 3 and baseline versus month 12 (for BCVA). RPE lesion area increased significantly from baseline to month 3 (*P* = 0.0039) and subsequently decreased from month 3 to month 12 (*P* = 0.0039). BCVA improved significantly, reflected by lower LogMAR values, from baseline to month 3 (*P* = 0.0156) and from baseline to month 12 (*P* = 0.0039). Asterisks indicate statistical significance: *P < 0.05; **P < 0.01. Because some eyes had identical BCVA values at certain visits (e.g., baseline and month 3), individual trajectories may partially overlap in panel **(B)** However, all 10 distinct trajectories are fully distinguishable in the month 6 to month 9 interval where no identical overlapping values occur.

Among eyes with chorioretinal involvement included in the longitudinal RPE lesion-area analysis (n = 10), RPE lesion area and BCVA followed different time courses during follow-up (**Figure 3**). Overall, significant longitudinal changes were observed for both RPE lesion area and BCVA (summarized in **Supplementary Table 1**). For RPE lesion area, a significant increase was observed from baseline to month 3 (*P* = 0.0039), followed by a significant decrease from month 3 to month 12 (*P* = 0.0039). In contrast, BCVA showed improvement, with a significant decrease in LogMAR from baseline to month 3 (*P* = 0.0156), and remained significantly improved at month 12 compared with baseline (*P* = 0.0039). Detailed pairwise comparison *P* values for RPE lesion area and BCVA are provided in **Supplementary Tables 2**, **3**, respectively.

We additionally explored whether the spatial extent of RPE damage was associated with final visual outcome. In the chorioretinal group (n = 10), Spearman correlation analysis did not show a statistically significant monotonic correlation between RPE lesion area and 12-month LogMAR BCVA (baseline area: *r_s_* = 0.061, *P* = 0.866; month-3 area: *r_s_* = 0.120, *P* = 0.742).

### Postoperative complications and disease course

**Figure 4:**
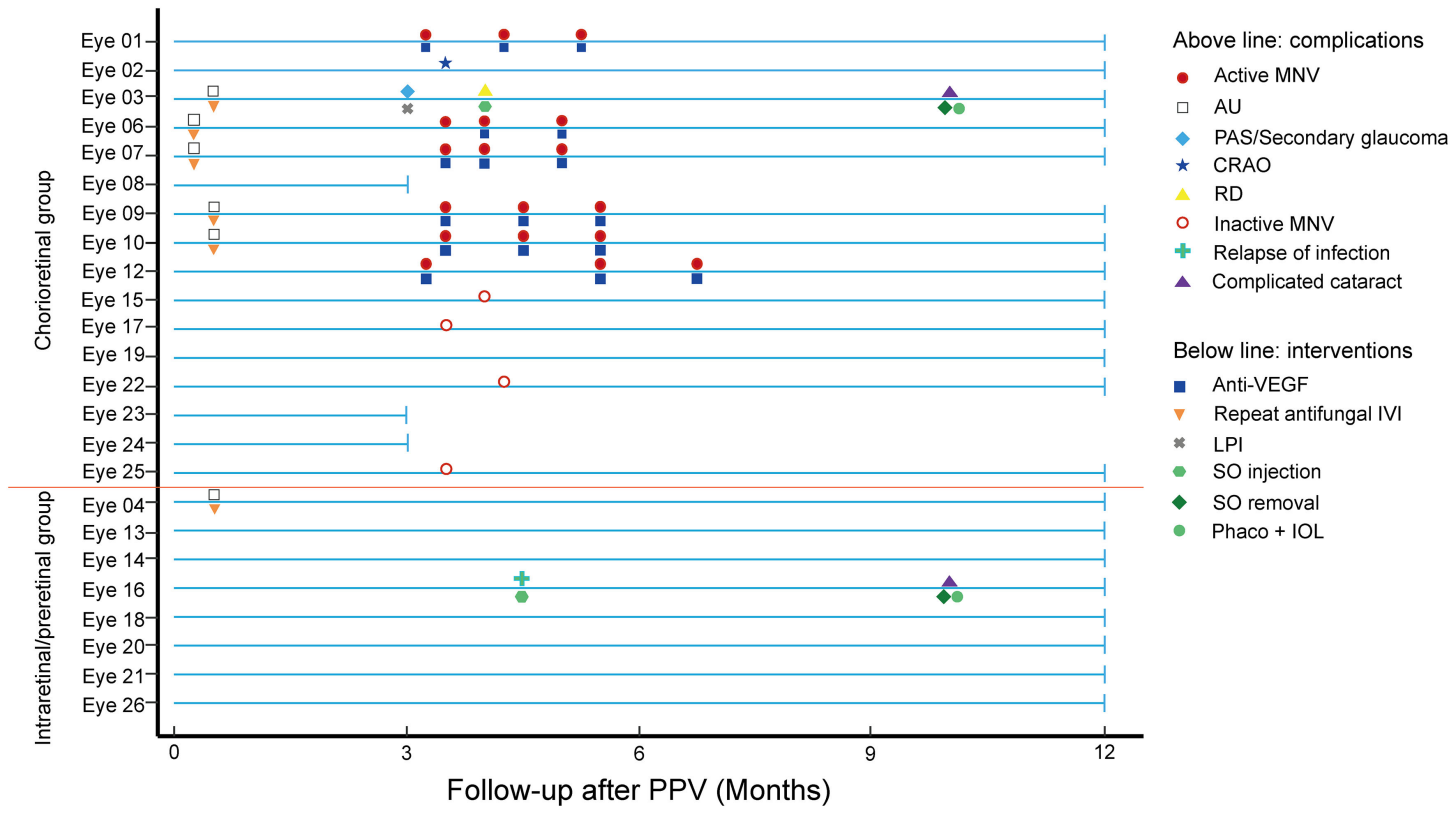
Swimmer plot of disease course, postoperative events, and subsequent interventions during follow-up. The swimmer plot summarizes the clinical course of individual eyes in the chorioretinal group (n = 13 eyes) and the intraretinal/preretinal group (n = 8 eyes) over 12 months after pars plana vitrectomy (PPV). Each horizontal line represents one eye, with month 0 defined as the day of PPV. Symbols above each timeline indicate postoperative events, and symbols below each timeline indicate subsequent interventions or surgical procedures. The orange horizontal divider separates the two lesion-pattern groups. Filled and open red circles denote active and inactive MNV, respectively. Anti-VEGF, anti–vascular endothelial growth factor; AU, anterior uveitis; CRAO, central retinal artery occlusion; IOL, intraocular lens; IVI, intravitreal injection; LPI, laser peripheral iridotomy; MNV, macular neovascularization; PAS, peripheral anterior synechiae; Phaco + IOL, phacoemulsification with intraocular lens implantation; PPV, pars plana vitrectomy; RD, retinal detachment; SO, silicone oil.

Postoperative complications and disease course are summarized in **Table 2**, **Figure 4**. During the 12-month follow-up period, eyes with chorioretinal involvement experienced a substantially higher overall complication burden than eyes with intraretinal/preretinal involvement. At least one postoperative complication occurred in 12 of 13 eyes (92.3%) in the chorioretinal group compared with 2 of 8 eyes (25.0%) in the intraretinal/preretinal group (*P* < 0.001).

**Figure 5:**
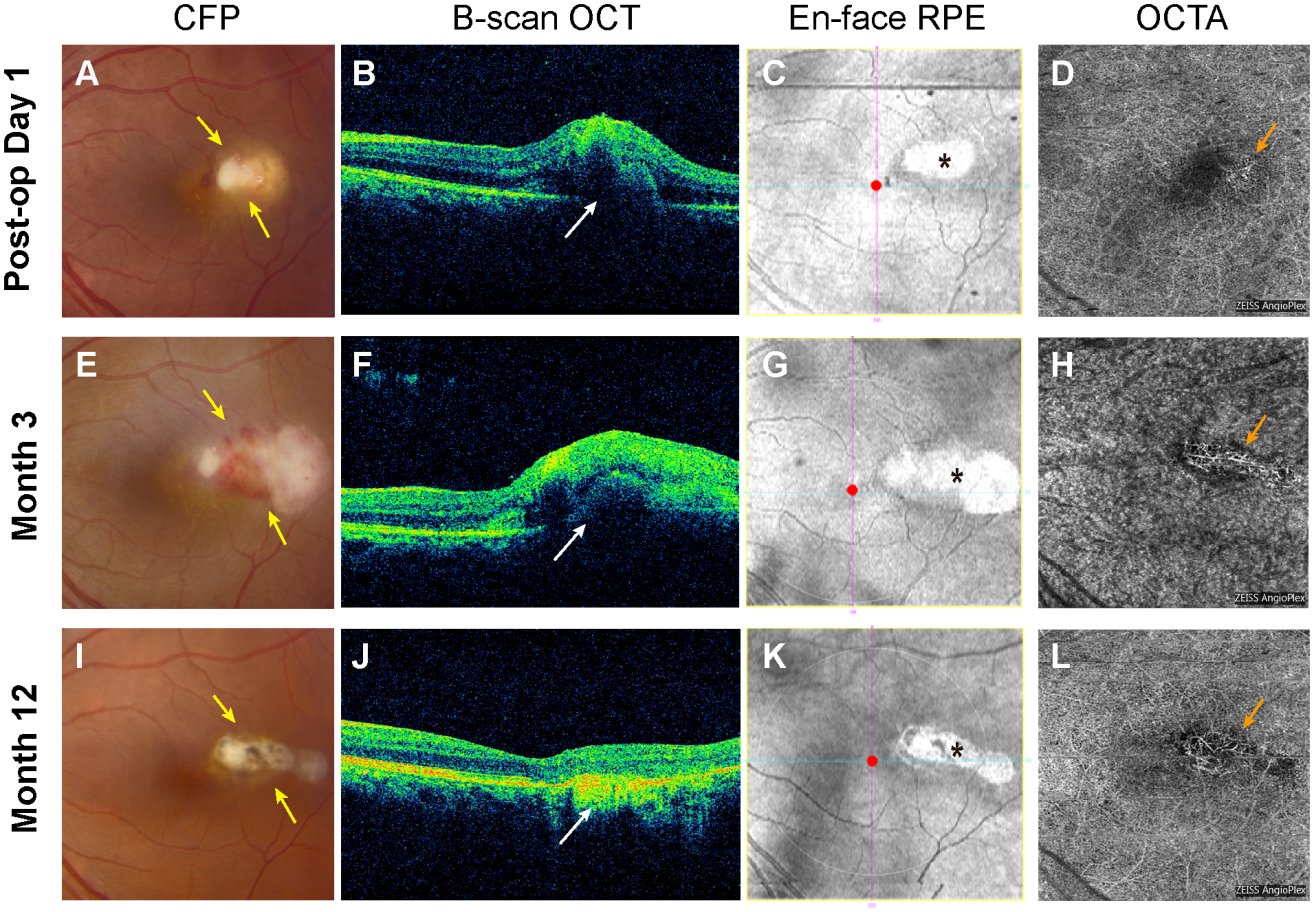
Representative longitudinal morphological changes of a macular lesion in a patient from the chorioretinal group. Multimodal imaging, including color fundus photography (CFP; first column), B-scan optical coherence tomography (OCT; second column), en-face retinal pigment epithelium (RPE) mapping (third column), and OCT angiography (OCTA; fourth column), demonstrates the progression of the lesion at postoperative day 1 **(A–D)**, month 3 **(E–H)**, and month 12 **(I–L)**. Yellow arrows on CFP indicate the superior and inferior margins of the chorioretinal lesion, visualizing its initial presentation, marked expansion at month 3, and the subsequent fibrotic contraction process at month 12, which is accompanied by clear demarcation, pigmentation, and scarring. White arrows on B-scan OCT point to the subretinal hyperreflective lesion. B-scan OCT shows a subretinal hyperreflective lesion with posterior shadowing on postoperative day 1 and month 3, which evolved into a dense hyperreflective scar with increased posterior signal transmission by month 12. Black asterisks (*) on en-face RPE images denote the corresponding hyperreflective area of the RPE lesion, which parallels the quantitative biphasic area changes described in **Figure 3**. Orange arrows on OCTA highlight the vascular alterations; notably, at postoperative day 1 **(D)**, the arrow indicates choriocapillaris alteration without a definite neovascular network, whereas at month 3 **(H)** and month 12 **(L)**, the arrows point to the distinct formation and evolution of a MNV network.

MNV was the most frequent vision-threatening complication in eyes with chorioretinal involvement. Among eyes with evaluable longitudinal retinal imaging, secondary MNV developed in 10 of 10 eyes in the chorioretinal group (100.0%; 95% CI, 69.2%–100.0%), whereas no eye in the intraretinal/preretinal group developed MNV (0.0%; 95% CI, 0.0%–36.9%; *P* < 0.001). Given the small denominator, these wide confidence intervals statistically highlight the underlying uncertainty, and this uniform 100.0% occurrence should be interpreted with caution. Among the 10 eyes that developed secondary MNV in the chorioretinal group, 6 eyes (60.0%) presented with active MNV and underwent anti-VEGF therapy, while the remaining 4 eyes (40.0%) presented with inactive MNV and were monitored without treatment. All 6 treated eyes completed the recommended 3-dose monthly loading regimen of conbercept, and none required additional PRN injections during the 12-month follow-up as the lesions subsequently stabilized and transitioned to fibrotic scars. The mean number of injections per treated eye was 3.0 ± 0.0 (range, 3–3). A representative case illustrating the typical longitudinal evolution of a chorioretinal lesion is shown in **Figure 5**. By month 3, the RPE lesion area had enlarged and a secondary MNV network was detected on OCTA. By month 12, the lesion showed fibrotic contraction and dense chorioretinal scarring, with persistent MNV. Other vision-threatening complications were less frequent. Retinal detachment and central retinal artery occlusion each occurred in 1 eye (7.7%) in the chorioretinal group and in no eyes in the intraretinal/preretinal group. Relapse of infection occurred in 1 eye (12.5%) in the intraretinal/preretinal group and in no eyes in the chorioretinal group.

Other ocular complications included anterior uveitis, complicated cataract, and peripheral anterior synechiae/secondary glaucoma. Anterior uveitis occurred in 5 eyes (38.5%) in the chorioretinal group and 1 eye (12.5%) in the intraretinal/preretinal group (*P* = 0.354). Complicated cataract occurred in 1 eye in each group, and peripheral anterior synechiae/secondary glaucoma occurred in 1 eye in the chorioretinal group.

The swimmer plot illustrates the timing of postoperative events and subsequent interventions for individual eyes over 12 months after PPV (**Figure 4**). In the chorioretinal group, multiple eyes required anti-VEGF therapy for active MNV during follow-up. Other secondary interventions during follow-up included repeat intravitreal antifungal injection and laser peripheral iridotomy. Notably, two eyes required secondary vitrectomy with silicone oil tamponade at approximately month 4 (one for postoperative retinal detachment and one for recurrent endophthalmitis). Both of these eyes subsequently underwent combined silicone oil removal and phacoemulsification with intraocular lens implantation for complicated cataract approximately 6 months later (around month 10).

## Discussion

In this study, we characterized the clinical trajectories of a unique cohort of immunocompetent patients who developed infusion-related endogenous *Candida albicans* endophthalmitis based on initial lesion morphology. Unlike the vast majority of previous reports which predominantly describe the disease in immunocompromised individuals or those with severe systemic comorbidities (e.g., ICU patients, post-surgical sepsis) (Schiedler et al., 2004; Sallam et al., 2012), our cohort stemmed from a rare outbreak attributed to contaminated infusions in otherwise healthy patients. This setting allowed us to examine ocular disease progression with fewer systemic confounders than in typical EFE series, where immunosuppression and critical illness are common.

Our main finding was that, although both groups experienced visual improvement after treatment, eyes with chorioretinal involvement had worse final BCVA, more postoperative complications, and a higher frequency of secondary MNV than eyes with intraretinal/preretinal involvement. These findings indicate that chorioretinal involvement identifies a subgroup requiring closer structural monitoring. This pattern is consistent with previous reports regarding the prognostic value of lesion location (Schiedler et al., 2004; Sallam et al., 2012), while also providing a less confounded view of ocular disease progression after *Candida* endophthalmitis.

Our two groups were comparable in baseline demographics, initial inflammation severity, and surgical timing, suggesting that these factors were unlikely to fully account for the different outcomes. Rather, the main difference between groups was the depth of tissue involvement. While infectious lesions might overlie the macula topographically in either group, OCT-confirmed structural disruption of the macular outer retina, RPE, or choroid was exclusively observed in the chorioretinal group. This suggests that the depth of initial tissue involvement may be an important determinant of long-term visual outcomes. The poorer prognosis in the chorioretinal group may therefore be explained, at least in part, by structural damage rather than by the topographic proximity of lesions to the macula alone. The macula’s central role, particularly when its outer layers are compromised, is a well-known predictor of poor visual prognosis, and our data extends this understanding to cases of infectious origin (Schiedler et al., 2004; Sallam et al., 2012).

A clinically important finding was the high frequency of secondary MNV in the chorioretinal group. Notably, secondary MNV was detected in all chorioretinal eyes with gradable longitudinal macular imaging, whereas no MNV was observed in the intraretinal/preretinal group. This pattern is consistent with the anatomical proximity of the infection to the RPE–Bruch membrane–choriocapillaris complex.

We hypothesize that hematogenous *Candida* seeding involving the choroidal circulation may disrupt the outer blood–retinal barrier and Bruch membrane–RPE complex. This structural compromise, combined with post-infection inflammation, local hypoxia, and tissue repair pathways, may contribute to a local pro-angiogenic environment that favors secondary neovascularization (Lodhi et al., 2017; D’cruz et al., 2025; Servillo et al., 2025). However, these imaging-based observations must be presented as biologically plausible hypotheses rather than proven molecular mechanisms, as the precise cellular pathways of active tissue repair in EFE remain to be fully elucidated. This delayed angiogenic process is illustrated in **Figure 5**, where early choriocapillaris alteration without a definite neovascular network on postoperative day 1 evolved into a definite MNV network by month 3.

In contrast, the absence of secondary MNV in the intraretinal/preretinal group may reflect relative preservation of the Bruch membrane–RPE barrier. Because these lesions were confined to the inner retina or vitreoretinal interface, they may have spared the RPE–Bruch membrane–choriocapillaris complex and were therefore less likely to trigger choroid-derived neovascularization. Given the limited sample size, we interpret this finding cautiously and consider it hypothesis-generating. Studying this process in an immunocompetent cohort reduces the confounding effects of systemic immunosuppression and suggests that the underlying mechanisms may overlap with those described in other infectious or inflammatory chorioretinal diseases, such as viral retinitis and ocular tuberculosis (Lee Kim et al., 2017; Lodhi et al., 2017; D’cruz et al., 2025). A photo essay on endogenous *Candida* endophthalmitis also illustrates diverse ways neovascularization emerges from inflammation or RPE damage, aligning with our findings (Invernizzi et al., 2018). From a pathophysiological standpoint, the distinct lesion patterns observed on OCT likely reflect different stages or routes of host-pathogen interactions. *Candida albicans* possesses a unique capacity for yeast-to-hyphae transition, which facilitates active tissue invasion and incites a potent host inflammatory response (Gow et al., 2011). When the fungal load primarily seeds the vitreous or inner retina, it manifests as vitreous “puff-balls” or superficial intraretinal infiltrates with a relatively preserved outer retina. Conversely, when hematogenous seeding involves the choriocapillaris, progressive inflammatory infiltration leads to localized necrosis of the RPE and Bruch’s membrane, which we captured as deep chorioretinal lesions on OCT (Edwards et al., 1974). This clinical distribution reinforces the concept that structural compromise of the outer blood-retinal barrier—rather than the absolute spatial dimension of the initial lesion—serves as a primary permissive factor for secondary neovascularization.

We also observed divergent time courses between RPE lesion area and BCVA in the chorioretinal group. As illustrated in **Figure 5**, the RPE lesion area increased during the first 3 months and subsequently decreased by month 12, whereas BCVA improved early and remained better than baseline. This suggests that an initial increase in RPE lesion area should not be misidentified as an active or ongoing infection. Instead, this expansion likely reflects progressive lesion demarcation, localized scar contraction, and clearer visualization of RPE damage as the overlying optical media clears. Furthermore, the RPE–Bruch membrane complex may function as more than a simple structural marker, since its disruption and repair likely contribute to the local microenvironment promoting secondary MNV development (Lee Kim et al., 2017; Servillo et al., 2025). Consequently, an early expansion of RPE loss should be interpreted in conjunction with functional visual recovery rather than as an isolated indicator of treatment failure or disease progression.

Importantly, this transient enlargement of the RPE lesion boundary during the first 3 months did not lead to infectious recurrence or inflammatory relapse in any of the chorioretinal eyes. This clinical safety is further supported by the complete absence of new inflammatory vitreous haze, stable postoperative intraocular pressure, and sustained control of fungal activity achieved through early PPV and systemic voriconazole therapy. Therefore, postoperative surveillance should focus on functional and structural signs of active fluid exudation on OCT or vascular flow signals on OCTA, rather than relying solely on the anatomical footprint of RPE loss to suspect recurrence.

Clinically, initial lesion morphology may help stratify the risk of long-term structural complications after *Candida* EFE. Eyes with chorioretinal involvement require close OCT and OCTA surveillance for secondary MNV even after apparent infection control. However, the single recurrence observed in the intraretinal/preretinal group indicates that superficial lesion morphology does not eliminate the need for continued infection surveillance. Thus, although lesion depth appeared to be more closely related to structural sequelae such as MNV than to relapse risk in this small cohort, both neovascular complications and infectious recurrence should be considered during follow-up (Baxter et al., 2013; Servillo et al., 2025). To mitigate this risk, postoperative monitoring should incorporate routine structural OCT and OCTA to detect neovascular activity, which may develop with minimal clinical signs. OCTA is particularly useful for detecting inflammatory MNV (Agarwal et al., 2018; Aggarwal et al., 2019; Tang et al., 2020; Kongwattananon et al., 2022; Bou Ghanem et al., 2023; Servillo et al., 2025). When active secondary MNV is identified, individualized anti-vascular endothelial growth factor (anti-VEGF) therapy is often necessary. Although anti-VEGF therapy manages the angiogenic complications, PPV and antifungal treatments remain essential for controlling the underlying infection (Lee Kim et al., 2017; Servillo et al., 2025). This approach is consistent with current management principles for inflammatory MNV (Dhingra et al., 2010; Agarwal et al., 2018; Servillo et al., 2025).

Our study has several limitations. First, the sample size was small. This reflects the rapid identification of the outbreak and cessation of contaminated infusions, which prevented further exposure but limited statistical power and precluded definitive causal inference. Nevertheless, the single-source nature of the cohort reduced heterogeneity in pathogen source and host status, allowing a more focused assessment of ocular disease progression with fewer confounding systemic comorbidities than in typical EFE series. Another limitation relates to the timing of lesion-pattern classification. Because media opacity often precluded clear preoperative OCT visualization, initial lesion classification partially relied on postoperative day-1 OCT. This introduces the theoretical possibility that some observed outer retinal or RPE disruption could have been influenced by surgical manipulation. To reduce this risk, lesion manipulation was minimized when possible, and classification was based on postoperative OCT findings interpreted in conjunction with intraoperative observations. Additionally, although GEE models were used to account for inter-eye correlation in patients with bilateral involvement, statistical power remained limited. Similarly, the 100% incidence of secondary MNV among evaluable chorioretinal eyes should be interpreted cautiously because of the small denominator and wide confidence interval. Nevertheless, the absence of MNV in the intraretinal/preretinal group suggests a clinically meaningful difference between the two lesion patterns. Furthermore, we did not quantify longitudinal retinal/choroidal thickness or macular edema volume, because dense vitreous haze and severe outer retinal/RPE structural disruption in the acute phase frequently caused unreliable automated OCT segmentation; attempting manual segmentation in these heavily damaged areas would introduce substantial subjective bias. The retrospective exclusion of eyes with advanced vitreous destruction or early complications (such as primary retinal detachment or CRAO) at baseline represents a potential selection bias, which might have biased our longitudinal functional results toward more favorable visual outcome estimates. While the single-center retrospective design limits the generalizability of our findings to other clinical settings, it minimized variability in surgical techniques, treatment protocols, and multimodal imaging acquisition. Future multicenter prospective studies using standardized imaging protocols are needed to confirm the incidence and risk factors for secondary MNV. Finally, longer follow-up is needed to determine whether MNV activity after 12 months continues, recurs, or stabilizes.

In conclusion, initial lesion morphology was strongly associated with visual prognosis in immunocompetent patients with infusion-related *Candida albicans* EFE. Chorioretinal involvement was associated with structural macular damage, poorer visual recovery, extensive RPE remodeling, and a higher risk of secondary MNV. These findings support close multimodal monitoring and timely treatment of active secondary MNV to optimize visual outcomes.

## Data Availability

The original contributions presented in the study are included in the article/**Supplementary Material**. Further inquiries can be directed to the corresponding author.

## References

[B1] Agarwal A. Invernizzi A. Singh R. B. Foulsham W. Aggarwal K. Handa S. . (2018). An update on inflammatory choroidal neovascularization: Epidemiology, multimodal imaging, and management. J. Ophthal Inflamm. Infect. 8, 13. doi: 10.1186/s12348-018-0155-6 30209691 PMC6135736

[B2] Aggarwal K. Agarwal A. Sharma A. Sharma K. Gupta V. OCTA Study Group (2019). Detection of type 1 choroidal neovascular membranes using optical coherence tomography angiography in tubercular posterior uveitis. Retina 39, 1595–1606. doi: 10.1097/IAE.0000000000002176 29689028

[B3] Baxter S. L. Pistilli M. Pujari S. S. Liesegang T. L. Suhler E. B. Thorne J. E. . (2013). Risk of choroidal neovascularization among the uveitides. Am. J. Ophthalmol. 156, 468–477.e2. doi: 10.1016/j.ajo.2013.04.040 23795984 PMC3748230

[B4] Bihaniya H. Rudraprasad D. Joseph J. (2024). Pathobiology of fungal endophthalmitis: A major review. ACS Infect. Dis. 10, 3126–3137. doi: 10.1021/acsinfecdis.4c00442 39267469

[B5] Bou Ghanem G. Neri P. Dolz-Marco R. Albini T. Fawzi A. (2023). Review for diagnostics of the year: Inflammatory choroidal neovascularization – imaging update. Ocular Immunol. Inflammation 31, 819–825. doi: 10.1080/09273948.2022.2046793 35404739

[B6] Burton E. Reddy V. Venkat A. G. (2024). Endogenous fungal endophthalmitis: A single-center retrospective study and review of the literature. Am. J. Ophthalmol. 262, 97–106. doi: 10.1016/j.ajo.2024.01.018 38280676

[B7] Chen K.-J. Sun M.-H. Chen Y.-P. Chen Y.-H. Wang N.-K. Liu L. . (2022). Endogenous fungal endophthalmitis: Causative organisms, treatments, and visual outcomes. JoF 8, 641. doi: 10.3390/jof8060641 35736124 PMC9225322

[B8] D’cruz R. Anjana T. Shaji M. (2025). Type 1 inflammatory choroidal neovascular membrane in a case of viral uveitis - a case report. Int. J. Surg. Case Rep. 134, 111650. doi: 10.1016/j.ijscr.2025.111650 40782457 PMC12355129

[B9] Danielescu C. Anton N. Stanca H. T. Munteanu M. (2020). Endogenous endophthalmitis: A review of case series published between 2011 and 2020. J. Ophthalmol. 2020, 1–13. doi: 10.1155/2020/8869590 33149945 PMC7603614

[B10] Das T. Agarwal M. Anand A. R. Behera U. C. Bhende M. Das A. V. . (2022). Fungal endophthalmitis. Ophthalmol. Retina 6, 243–251. doi: 10.1016/j.oret.2021.09.006 34547530

[B11] Dhingra N. Kelly S. Majid M. Bailey C. Dick A. (2010). Inflammatory choroidal neovascular membrane in posterior uveitis-pathogenesis and treatment. Indian J. Ophthalmol. 58, 3. doi: 10.4103/0301-4738.58467 20029141 PMC2841372

[B12] Dogra M. Akella M. Dogra M. R. Gupta A. (2018). Presumably contaminated intravenous infusion-induced Aspergillus terreus endogenous endophthalmitis presenting with posterior hypopyon. Indian J. Ophthalmol. 66, 593–595. doi: 10.4103/ijo.IJO_695_17 29582833 PMC5892075

[B13] Edwards J. E. Foos R. Y. Montgomerie J. Z. Guze L. B. (1974). Ocular manifestations of candida septicemia: Review of seventy-six cases of hematogenous Candida endophthalmitis. Med. (Baltimore) 53, 47–75. doi: 10.1097/00005792-197401000-00002 4587001

[B14] Gow N. A. van de Veerdonk F. L. Brown A. J. Netea M. G. (2011). Candida albicans morphogenesis and host defence: Discriminating invasion from colonization. Nat. Rev. Microbiol. 10, 112–122. doi: 10.1038/nrmicro2711 22158429 PMC3624162

[B15] Gupta A. Gupta V. Dogra M. R. Chakrabarti A. Ray P. Ram J. . (2000). Fungal endophthalmitis after a single intravenous administration of presumably contaminated dextrose infusion fluid. Retina 20, 262–268. doi: 10.1097/00006982-200003000-00007 10872931

[B16] Invernizzi A. Cozzi M. Symes R. Pellegrini M. Staurenghi G. (2018). Ocular neovascularization in endogenous Candida endophthalmitis: Using multimodal imaging to understand different pathogenic pathways. Retina 38, e17–e19. doi: 10.1097/IAE.0000000000001978 29190230

[B17] Karkhur S. Afridi R. Menia N. Gupta N. Nguyen Q. D. Dogra M. . (2020). Posterior hypopyon in fungal endogenous endophthalmitis secondary to presumably contaminated dextrose infusion. Am. J. Ophthalmol. Case Rep. 18, 100681. doi: 10.1016/j.ajoc.2020.100681 32373756 PMC7191180

[B18] Kongwattananon W. Grasic D. Lin H. Oyeniran E. Sen H. N. Kodati S. (2022). Role of optical coherence tomography angiography in detecting and monitoring inflammatory choroidal neovascularization. Retina 42, 1047–1056. doi: 10.1097/IAE.0000000000003420 35067607 PMC9124680

[B19] Lee Kim E. Rodger D. C. Rao N. A. (2017). Choroidal neovascularization secondary to tuberculosis: Presentation and management. Am. J. Ophthalmol. Case Rep. 5, 124–129. doi: 10.1016/j.ajoc.2016.12.025 29503964 PMC5758031

[B20] Lingappan A. Wykoff C. C. Albini T. A. Miller D. Pathengay A. Davis J. L. . (2012). Endogenous fungal endophthalmitis: Causative organisms, management strategies, and visual acuity outcomes. Am. J. Ophthalmol. 153, 162–166.e1. doi: 10.1016/j.ajo.2011.06.020 21917234

[B21] Lodhi S. A. K. Saifuddin K. Devulapally S. (2017). Inflammatory choroidal neovascular membrane after healed tuberculous choroidal granuloma. GMS Ophthalmol. cases 7, Doc06. doi: 10.3205/oc000057 28293535 PMC5340087

[B22] Sallam A. Taylor S. R. J. Khan A. McCluskey P. Lynn W. A. Manku K. . (2012). Factors determining visual outcome in endogenous candida endophthalmitis. Retina 32, 1129–1134. doi: 10.1097/IAE.0b013e31822d3a34 22298012

[B23] Schiedler V. Scott I. U. Flynn H. W. Davis J. L. Benz M. S. Miller D. (2004). Culture-proven endogenous endophthalmitis: Clinical features and visual acuity outcomes. Am. J. Ophthalmol. 137, 725–731. doi: 10.1016/j.ajo.2003.11.013 15059712

[B24] Servillo A. Scandale P. Oldoni G. Begar P. G. Bandello F. Miserocchi E. . (2025). Inflammatory choroidal neovascularization: An evidence-based update. Survey Ophthalmol. 70, 451–466. doi: 10.1016/j.survophthal.2024.12.004 39706532

[B25] Shen X. Xu G. (2009). Vitrectomy for endogenous fungal endophthalmitis. Ocular Immunol. Inflammation 17, 148–152. doi: 10.1080/09273940802689396 19585356

[B26] Stephens J. D. Adam M. K. Todorich B. Faia L. J. Garg S. Dunn J. P. . (2017). Optical coherence tomography findings in endogenous fungal chorioretinitis, retinitis, and endophthalmitis. Ophthalmic Surg. Lasers Imaging Retina 48, 894–901. doi: 10.3928/23258160-20171030-04 29121358

[B27] Tanaka H. Ishida K. Yamada W. Nishida T. Mochizuki K. Kawakami H. (2016). Study of ocular candidiasis during nine-year period. J. Infect. Chemother. 22, 149–156. doi: 10.1016/j.jiac.2015.12.001 26778254

[B28] Tang W. Guo J. Liu W. Xu G. (2020). Optical coherence tomography angiography of inflammatory choroidal neovascularization early response after anti-VEGF treatment. Curr. Eye Res. 45, 1556–1562. doi: 10.1080/02713683.2020.1767790 32394732

[B29] Wang X. Zhang P. Suo J. Li Q. Zhang Y. (2025). The diagnosis and treatment progress of infectious endophthalmitis. Eye 39, 492–504. doi: 10.1038/s41433-024-03474-7 39616279 PMC11794455

[B30] Zhang X. Huang Y. Kang C. Zhang J. Zhong W. Xue J. . (2026). An outbreak of endogenous fungal endophthalmitis in immunocompetent individuals caused by presumed intravenous infusion contamination. Front. Cell. Infect. Microbiol. 16, 1718140. doi: 10.3389/fcimb.2026.1718140 42112464 PMC13153087

[B31] Zhang Y.-Q. Wang W.-J. (2005). Treatment outcomes after pars plana vitrectomy for endogenous endophthalmitis. Retina 25, 746–750. doi: 10.1097/00006982-200509000-00010 16141863

[B32] Zhuang H. Ding X. Gao F. Zhang T. Ni Y. Chang Q. . (2020). Optical coherence tomography features of retinal lesions in Chinese patients with endogenous Candida endophthalmitis. BMC Ophthalmol. 20, 52. doi: 10.1186/s12886-020-01337-9 32059661 PMC7020574

