# Supplementary material for: Twelve-month functional and structural outcomes of Candida endogenous endophthalmitis in immunocompetent patients: a longitudinal cohort study of an outbreak
Source: Front Cell Infect Microbiol. 2026 Jul 17;16:1901253. doi: 10.3389/fcimb.2026.1901253 (PMC13423705; doi:10.3389/fcimb.2026.1901253)
Supplement: Supplementary file 1 [file Table1.docx]

**Supplementary Table 1**

**Supplementary Table 1. Changes in RPE Lesion Area and Best-Corrected Visual Acuity Over 12 Months (n = 10)**

| **Parameter** | **Baseline** | **3 months** | **6 months** | **9 months** | **12 months** | ***P* value** |
| --- | --- | --- | --- | --- | --- | --- |
| **RPE Lesion Area (mm²)** | 1.68 ± 1.87 | 2.23 ± 2.41 | 1.78 ± 2.20 | 1.32 ± 1.40 | 1.33 ± 1.33 | 0.0010* |
| **BCVA (LogMAR)** | 1.62 ± 0.79 | 0.90 ± 0.49 | 0.71 ± 0.42 | 0.62 ± 0.42 | 0.72 ± 0.40 | 0.0008* |

Data are presented as mean ± standard deviation (SD) for 10 eyes with chorioretinal involvement.

BCVA = best-corrected visual acuity; LogMAR = logarithm of the minimum angle of resolution; RPE = retinal pigment epithelium.

* Indicates statistical significance (P < 0.05). P values represent the overall time effect across all visits, calculated using the Friedman test.

**Supplementary Table 2**

**Supplementary Table 2. Pairwise Comparisons of RPE Lesion Area Over 12 Months**

|  | **Baseline** | **3 months** | **6 months** | **9 months** | **12 months** |
| --- | --- | --- | --- | --- | --- |
| **Baseline** | **—** | 0.0039* | 0.8652 | 0.1250 | 0.1641 |
| **3 months** | 0.0039* | **—** | 0.0938 | 0.0078* | 0.0039* |
| **6 months** | 0.8652 | 0.0938 | **—** | 0.0156* | 0.1094 |
| **9 months** | 0.1250 | 0.0078* | 0.0156* | **—** | 0.6094 |
| **12 months** | 0.1641 | 0.0039* | 0.1094 | 0.6094 | **—** |

Data represent *P* values from two-sided Wilcoxon signed-rank tests for paired comparisons of RPE lesion area (mm²) between different time points (n = 10).

RPE = retinal pigment epithelium.

*****Statistically significant at *P* < 0.05.

**Supplementary Table 3**

**Supplementary Table 3. Pairwise Comparisons of Best-Corrected Visual Acuity Over 12 Months**

|  | **Baseline** | **3 months** | **6 months** | **9 months** | **12 months** |
| --- | --- | --- | --- | --- | --- |
| **Baseline** | **—** | 0.0156* | 0.0117* | 0.0020* | 0.0039* |
| **3 months** | 0.0156* | **—** | 0.2969 | 0.1250 | 0.3008 |
| **6 months** | 0.0117* | 0.2969 | **—** | 0.1172 | 0.6250 |
| **9 months** | 0.0020* | 0.1250 | 0.1172 | **—** | 0.0938 |
| **12 months** | 0.0039* | 0.3008 | 0.6250 | 0.0938 | **—** |

Data represent *P* values from two-sided Wilcoxon signed-rank tests for paired comparisons of BCVA (LogMAR) between different time points (n = 10).

BCVA = best-corrected visual acuity; LogMAR = logarithm of the minimum angle of resolution.

*Statistically significant at *P* < 0.05.
